# Artificial intelligence in Emergency Medical Services dispatching: assessing the potential impact of an automatic speech recognition software on stroke detection taking the Capital Region of Denmark as case in point

**DOI:** 10.1186/s13049-022-01020-6

**Published:** 2022-05-12

**Authors:** Mirjam Lisa Scholz, Helle Collatz-Christensen, Stig Nikolaj Fasmer Blomberg, Simone Boebel, Jeske Verhoeven, Thomas Krafft

**Affiliations:** 1 Emergency Medical Services, Capital Region of Denmark, Telegrafvej 5, 2750 Ballerup, Denmark; 2 Department of Health, Ethics and Society, Care and Public Health Research Institute (CAPHRI), Faculty of Health, Medicine and Life Sciences, Maastricht University, P.O. Box 616, 6200 MD Maastricht, Netherlands

**Keywords:** Artificial intelligence, Emergency Medical Services, Stroke detection, Automated speech recognition

## Abstract

**Background and purpose:**

Stroke recognition at the Emergency Medical Services (EMS) impacts the stroke treatment and thus the related health outcome. At the EMS Copenhagen 66.2% of strokes are detected by the Emergency Medical Dispatcher (EMD) and in Denmark approximately 50% of stroke patients arrive at the hospital within the time-to-treatment. An automatic speech recognition software (ASR) can increase the recognition of Out-of-Hospital cardiac arrest (OHCA) at the EMS by 16%. This research aims to analyse the potential impact an ASR could have on stroke recognition at the EMS Copenhagen and the related treatment.

**Methods:**

Stroke patient data (*n* = 9049) from the years 2016–2018 were analysed retrospectively, regarding correlations between stroke detection at the EMS and stroke specific, as well as personal characteristics such as stroke type, sex, age, weekday, time of day, year, EMS number contacted, and treatment. The possible increase in stroke detection through an ASR and the effect on stroke treatment was calculated based on the impact of an existing ASR to detect OHCA from CORTI AI.

**Results:**

The Chi-Square test with the respective post-hoc test identified a negative correlation between stroke detection and females, the 1813-Medical Helpline, as well as weekends, and a positive correlation between stroke detection and treatment and thrombolysis. While the association analysis showed a moderate correlation between stroke detection and treatment the correlation to the other treatment options was weak or very weak. A potential increase in stroke detection to 61.19% with an ASR and hence an increase of thrombolysis by 5% in stroke patients calling within time-to-treatment was predicted.

**Conclusions:**

An ASR can potentially improve stroke recognition by EMDs and subsequent stroke treatment at the EMS Copenhagen. Based on the analysis results improvement of stroke recognition is particularly relevant for females, younger stroke patients, calls received through the 1813-Medical Helpline, and on weekends.

***Trial registration*:**

This study was registered at the Danish Data Protection Agency (PVH-2014-002) and the Danish Patient Safety Authority (R-21013122).

## Background

According to the World Health Organization (WHO), strokes were the second leading cause of death and the third leading cause of disability-adjusted life years (DALYs) globally, in 2019 [1]. With 63.5 deaths per 100,000 population, stroke is among the top ten causes of death in Denmark [2]. Additionally, with 1024.1 DALYs per 100,000 population, strokes are within the top ten causes of DALYs in Denmark [3]. This is due to stroke patients irretrievably losing approximately 1.9 million neurons, every untreated minute after stroke onset, leading to 1.8 DALYs per minute [4, 5]. Several studies have determined that patients with a time-to-treatment of 90 min showed the best health outcome [6–9]. However, a benefit of intravenous thrombolysis with alteplase for patients with acute ischaemic stroke can be achieved within a time-to-treatment of 4.5 h [6, 9, 10]. Thus, it is crucial to minimize the time between stroke onset and treatment to reduce the mortality as well as the DALYs caused by strokes [11].

To initially get access to hospital treatment in Denmark, patients need to be referred to the hospital by a general practitioner or through the medical helplines (1-1-2 or 1813) of the Emergency Medical Services (EMS) [12]. While the 1-1-2 is the emergency number, the 1813-Medical Helpline (1813) serves as an out-of-hours number providing direct contact to specially trained nurses and physicians within the same emergency dispatch centre of the Capital Region of Denmark [13]. Previous research has shown, that the accurate and early stroke detection by the EMS plays an important role in the timely hospital admission of stroke patients, through dispatching a high priority ambulance (“A” response) [14–17]. Hsieh et al. [18] and Oostema et al. [19] have found that stroke detection by Emergency Medical Dispatchers (EMDs) leads to an improved stroke care and accordingly a better outcome for stroke patients [20]. However, several studies have shown that the accuracy of stroke detection among EMDs are highly variable, between 30 and 83% [8, 21, 22]. In Denmark, an observational study from 2012–2014 found a sensitivity of 66.2% in stroke recognition at the EMS Copenhagen [15]. Additionally, Amtoft et al. [23] identified, that approximately 50% of stroke patients in Denmark did not arrive at the hospital within the stated 4.5 h window of revascularization.

Previous research by Blomberg et al. [24] and Cleve et al. [25] has shown, that artificial intelligence (AI) in emergency medicine increases the accuracy as well as efficiency and reduces the time-to-treatment. Previously, an automatic speech recognition software (ASR) for the detection of Out-of-Hospital cardiac arrests (OHCA) by CORTI AI has proven to increase the sensitivity of OHCA from 72.5 to 84.1% and reduce the median time-to-recognition from 54 to 44 s at the EMS Copenhagen [24]. This software “listens” to the emergency call, processes the audio, transforms it into a textual representation, analyses it and outputs a prediction on the potential presence of cardiac arrest. Based on advanced speech analysis through AI, the technology structures and analyses all sounds and spoken information during a live EMS conversation and converts this data into a valid prediction [25]. The software continuously learns from previous patient consultations and published medical papers in the specific field of cardiac arrest [24, 25].

In line with the EU’s values to “become a global leader in innovation in the data economy and its application” [26] and considering the success of the ASR for OHCA by CORTI AI, the question arises, whether an ASR could improve the accuracy and speed of detection, and thus reduce the burden of disease, of other time-critical medical issues, like strokes [27]. Accordingly, this research aims to determine, *how an ASR, at the EMS Copenhagen, could contribute to a more accurate stroke detection and impact the stroke related treatment*. The following research questions address this aim:

- How many strokes are detected at the EMS Copenhagen and independently in the EMS numbers 1-1-2 and 1813, throughout 2016–2018?
- Is there a difference in stroke characteristics (e.g., stroke type) and patient specific characteristics (e.g., age, sex, time of day, and weekday) between strokes detected and strokes not detected at the EMS Copenhagen, throughout 2016–2018?
- Is there a correlation between stroke detection at the EMS Copenhagen and the treatment a stroke patient received, throughout 2016–2018?
- Which additional number of strokes could potentially be recognized at the EMS Copenhagen using an ASR?
- How could this additional number of strokes detected effect stroke treatment?

These analyses were performed to determine necessity of improving the stroke detection rate at the EMS Copenhagen and predict the possible impact of an ASR on stroke recognition as well as the influence on the stroke related treatment.

## Methods

This research is a descriptive retrospective quantitative single case study [28, 29].

### Setting

The research has been performed with data from the Capital Region of Denmark, with a population of 1.85 million (2020) in an area of 2563 km^2^ [30, 31]. In the Capital Region of Denmark the 1-1-2 emergency number and the 1813-Medical Helpline serve as contact points of the EMS [12]. The 1-1-2 and 1813 are part of one emergency medical dispatch centre and allow the assessment of severity of the callers medical condition and an according response independent of the number dialled, thus providing a single point of contact for patients seeking help for emergency and/or acute conditions [12]. While the 1-1-2 serves as an immediate emergency contact, the 1813 is considered an alternative for the GP operated by nurses during the out-of-hours times, between 4 p.m. and 8 a.m. as well as on the weekends. The internal and external validity for our study is ensured by including all the stroke data of the Capital Region of Denmark within the respected timeframe [32, 33].

### Data collection

Retrospective research is performed on existing data from 2016–2018 [34]. For 2016–2018, data of 15,258 stroke patients, with the Capital Region of Denmark as emergency site, were extracted from the Danish Stroke Registry, a nationwide clinical database [35]. Stroke patients within this study were distinguished by the types ischaemic and haemorrhagic stroke. This data was joined with the EMS contacts of the stroke patients based on the Danish Det Centrale Personregister (CPR), a personal identification number of Danish citizens, extracted from the EMS database. EMS contacts in the context are considered all contacts to the EMS (1-1-2/1813) by or on behalf of a patient. Only stroke patients with an EMS contact seven days prior or seven days post the onset of the stroke were included in this research, since first stroke symptoms can already occur up to seven days prior to the stroke [36]. For stroke patients with several EMS contacts (based on the CPR), only the contact closest to the onset of stroke was included in this analysis, since this contact is most likely to be the stroke related contact. Stroke patients that did not contact the 1-1-2 or the 1813 were excluded. Stroke contacts to the EMS were coded in “stroke relevant criteria”, “stroke nonrelevant criteria”, and “missing criteria” based on the criteria of the Danish Index for 1-1-2 and the 1813-Index for the 1813. The Danish Index and the 1813-Index guide the EMDs in assessing the urgency of the emergency situation [13]. The EMS contacts that had an indication of chapter A.26.03. (Suspected stroke, hemiparesis) and A.26.04. (Suspected stroke, reduced consciousness or dizziness) within the Danish Index were coded as “stroke relevant criteria” within this research. All other chapters were considered “stroke nonrelevant criteria”. The stroke contacts were coded as “missing criteria” when no criteria based on the Danish Index or the 1813-Index were indicated by the EMD. Additionally, to the variables mentioned before, the following characteristics were included: response plan priority of the EMS, age, sex, year, treatment options thrombolysis, reperfusion, thrombectomy, endovascular, or surgical treatment, incident occurrence on a weekday or the weekend, the EMS number called, and the time-to-call within the time-to-treatment for thrombolysis of 4.5 h.

### Outcome measures

The outcomes measured are the number of strokes detected at the EMS and respectively at the 1-1-2 and 1813, as well as the difference between detection when using the two EMS access phone numbers. Additionally, the change of stroke detection throughout 2016–2018, the difference in stroke detection among age, sex, stroke type, year, weekday or weekend, and time of day was determined. Furthermore, correlation between treatment of a stroke patient and detection of stroke through the EMD was analysed. Lastly, a prediction on the presumable number of additional strokes detected at the EMS with CORTI AI and the presumable related change in stroke treatment was made. For this research, strokes are considered detected by the EMS if the criteria of the Danish Index or the 1813-Index were stroke relevant and if a high priority ambulance (“A”) was dispatched, as this is the assigned stroke response in Denmark [14]. Accordingly, a stroke is considered as “not detected”, if the criteria of the Danish Index or the 1813-Index were stroke relevant, but no “A” response was dispatched or if the criteria of the respective Index were not stroke relevant. For the analysis within this study, the beforenamed outcomes were analysed for “strokes detected” compared to strokes with “missing criteria” and “strokes not detected”, separated into “stroke relevant criteria but no “A” response” and “stroke nonrelevant criteria”.

### Data analysis

To analyse the correlation between two categorical variables within this research, the Chi-Square test was used [37, 38]. This applies to the variables: EMS number (1-1-2/1813), stroke type (ischaemic/haemorrhagic), year (2016–2018), sex (male/female), weekday (Monday-Friday/Saturday-Sunday), treatment (yes/no), and the treatment options, thrombolysis (yes/no), reperfusion (yes/no), thrombectomy (yes/no), endovascular (yes/no), and surgical treatment (yes/no), in correlation with the “strokes detected”, “strokes nonrelevant criteria”, “stroke relevant criteria but no “A” response”, and strokes with “missing criteria”. To avoid type I error, the Fisher’s Exact test was used for the analysis of characteristics with a cell frequency of < 5, such as the correlation between stroke recognition by the EMD and reperfusion, surgical as well as endovascular treatment [38, 39]. For the further analysis of the correlation analysed with the Fisher’s Exact test, the hereafter described analyses for the Chi-Square test were performed. To determine the goodness of fit a Log Likelihood Ratio was performed [40]. The goodness of fit was determined for all the named variables, with a statistically significant p-value of *p* ≤ 0.05 (95% CI) [40]. The strength of determined correlations was tested through an association analysis [38]. Within this, the Cramer’s V was interpreted, since it is considered a robust test for strength of association within multiple group studies [41]. Lastly, a post-hoc test for independence with adjusted residuals was performed based on a pairwise comparison with a Bonferroni-Holm correction, to adjust the significance level for variables with more than two characteristics [38, 42, 43].

An analysis of variance was performed for the interval scaled variables age and time of day. To respectively choose the suitable statistical test, the normality, using the Shapiro–Wilk test, and the homogeneity of variance, using the Levene’s test, were tested for the named variables among the four groups, “stroke detected”, “stroke nonrelevant criteria”, “stroke relevant criteria but no “A” response”, and “missing criteria” [44–46]. Since the Shapiro–Wilk test showed no normal distribution in any of the groups for age or time of day, the Kruskal–Wallis test was chosen to analyse the beforenamed correlations. The Kruskal–Wallis test was solely chosen due to no normal distribution of the stroke patients, however not according to the size of the groups. Additionally, the Wilcoxon–Mann–Whitney test with a Bonferroni-Holm correction was conducted as post-hoc test to determine the correlation between the individual groups through a pairwise comparison [47]. This post-hoc test was respectively chosen, due to the statistically significant Levene’s test (*p* < 0.05, 95% CI) determining no homogeneity of variance for the age of the stroke patients and time of call throughout the groups analysed [46].

The results for all analyses were considered statistically significant, when *p* ≤ 0.05 (95% CI), or based on the adjusted significance level within the post-hoc test of independence. The statistical analysis was performed with the statistical software R 3.6.3 [48].

Under the condition that the rise of the detection rate for strokes would be the same as the increase in OHCA detection rate through CORTI AI, the presumable increase of the detection rate of strokes using an ASR was calculated. This calculation was conducted based on the results of the analysis of strokes detected by the EMS. The following calculations were performed:

- *Detection rate of stroke with an ASR* = (Detection rate of OHCA with an ASR/Detection rate of OHCA without an ASR) * Detection rate of strokes at EMS
- *Strokes detected with an ASR* = Stroke patients calling EMS * Detection rate of strokes with an ASR
- *Additional strokes detected with an ASR* = *Strokes detected with an ASR − Strokes detected without an ASR*

Based on the results of this calculation, the potential change in treatment of stroke patients affected by stroke detection through the ASR was determined, under the condition that the number of strokes with “missing criteria” will not be influenced by the ASR. This condition is necessary, due to a lack of information on “missing criteria”. The following calculations were performed for the total amount of treatment as well as for each individual treatment:

- Strokes* with treatment*_*x*_* with an ASR* = *Strokes not detected through an ASR with treatment*_*x*_ + *Strokes detected through an ASR with treatment*_*x*_ + *Strokes “missing criteria” with treatment*_*x*_
- Additional* Strokes with treatment*_*x*_* with an ASR* = *Strokes with treatment*_*x*_* with an ASR* − *Strokes with treatment*_*x*_* without an ASR*
- *Treatment*_*x*_* Rate with an ASR* = *Strokes with *treatment_x_* with an ASR/Strokes*
- Change* in treatment*_*x*_* Rate with an ASR* = *(Treatment*_*x*_* Rate with an ASR/Treatment*_*x*_* Rate without an ASR)* − 1

The same analysis was performed for the subgroup of stroke patients calling within the time-to-treatment (4.5 h) of thrombolysis (*n* = 6013), as a sensitivity analysis to enable more precise predictions [49, 50]. Within the subgroup analysis the Fisher’s Exact test was additionally used for the analysis of thrombectomy due to cell frequencies < 5 [38, 39].

## Results

**Fig. 1:**
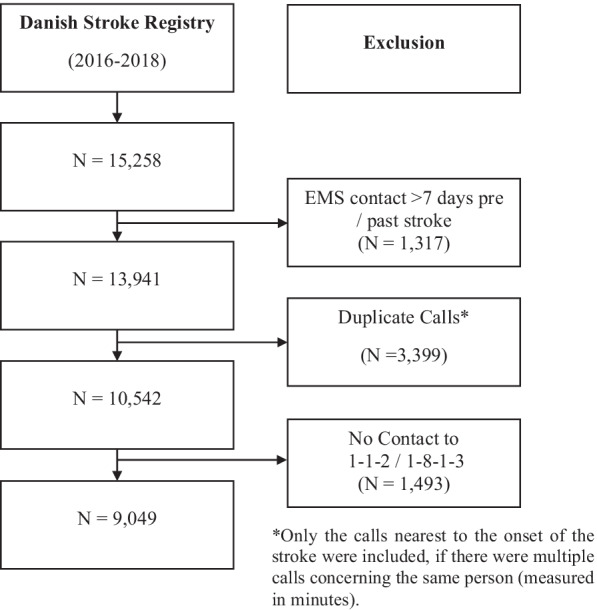
Consort flow chart in EMS of the Capital Region of Denmark 2016–2018

For the timeframe 2016–2018, 15,258 stroke patients from the Danish Stroke Registry within the Capital Region of Denmark were included in this research (Fig. 1) [35]. Based on the EMS database, the number of stroke related EMS contacts prior or post seven days of stroke within the respected timeframe were 13,941. 3399 duplicate EMS contacts for the same stroke patient and 1493 stroke patients without contact to the EMS were excluded. Finally, this resulted in the inclusion of 9049 stroke related EMS contacts. Baseline characteristics of the stroke patients included in this research, such as stroke type, sex, age, year, time of day, weekday, stroke relevant criteria, EMS response, EMS number, received treatment, and time-to-call can be found in Table 1.

**Table 1:** Baseline characteristics stroke patients in EMS of the Capital Region of Denmark 2016–2018

Characteristic	N	%
Stroke patients	9049	100.00
Type		
Ischaemic stroke	8215	90.78
Haemorrhagic stroke	834	9.22
Sex		
Male	4827	53.34
Female	4222	46.66
Age—mean (Min.|Q1|Median|Q3|Max.)	71.28 (18|63|73|81|104)	
EMS Number		
1-1-2	5180	57.24
1813-Medical Helpline	3869	42.76
Stroke criteria		
Stroke relevant criteria	5973	66.01
Stroke nonrelevant criteria	1861	20.57
Missing criteria	1215	13.43
Response [53]		
A (life threatening)	6128	67.72
B (urgent, no immediate risk to life)	1028	11.36
C (non-urgent/planned transport)	24	0.27
D (no treatment/medical care required)	4	0.04
F (referral/advice)	45	0.50
Non-Urgent Response*	1820	20.11
Year		
2016	2807	31.02
2017	3021	33.38
2018	3221	35.59
Weekday (Monday–Friday)	5854	64.69
Weekend (Saturday–Sunday)	3195	35.31
Incident time of day		
1–6	677	7.48
7–12	3542	39.14
13–18	3096	34.21
19–0	1734	19.16
Treatment^†^	1652	18.26
Thrombolysis	1449	16.01
Reperfusion	217	2.40
Endovascular	47	0.52
Surgical	43	0.48
Thrombectomy	239	2.64
Time-to-call		
≤ 4.5 h	6013	66.45
≤ 24 h	8095	89.46

*A patient with a “Non-Urgent Response” typically received a referral to a physician or was advised to drive to the hospital

^†^A patient who received multiple treatments was considered individually for the individual treatments but cumulated for the total number of treatments

### Outcomes

The results of the Chi-Square and Fisher’s Exact test showed a correlation to minimum one of the four groups of stroke recognition within all the considered variables, based on the determined level of significance *p* < 0.05 (Table 2). The standardised residuals show the direction of correlation and were generated within the pairwise comparison and interpreted in relation to the critical *z*-value, calculated based on the adjusted significance level [37, 51].

**Table 2:** Outcome Chi-Square test

Characteristic	Stroke detected			Stroke not detected					
	Stroke relevant criteria + “A” response			Stroke relevant criteria + no “A” response			Stroke nonrelevant criteria		
	*N*	*%*	Residuals	*N*	*%*	Residuals	*N*	*%*	Residuals
Total	4773 *3613*	52.75% *60.09%*	–	1200 *588*	13.26% *9.78%*	–	1861 *1134*	20.57% *18.86%*	–
Type									
Ischaemic	4345 *3288*	48.02% *54.68%*	0.8664729 *1.730522*	1117 *543*	12.34% *9.03%*	2.9572745 ***1.631358***	1629 *981*	18.00% *16.31%*	− 5.4382222 − *5.045376*
Haemorrhagic	428 *325*	4.73% *5.40%*	− 0.8664728 − *1.730522*	83 *45*	0.92% *0.75%*	− 2.9572745 − ***1.631358***	232 *153*	2.56% *2.54%*	5.4382222 *5.045376*
Sex									
Male	2663 *2008*	29.43% *33.39%*	4.9358538 *3.834136*	628 *294*	6.94% *4.89%*	− 0.7527075 − *1.826033*	950 *586*	10.50% *9.75%*	− 2.2267499 − *1.418067*
Female	2110 *1605*	23.32% *26.68%*	− 4.9358538 − *3.834136*	572 *294*	6.32% *4.89%*	0.7527075 *1.826033*	911 *548*	10.07% *9.11%*	2.2267499 *1.418067*
EMS number									
1-1-2	3566 *2761*	39.41% *45.92%*	35.486025 *23.923099*	427 *279*	4.72% *4.64%*	− 16.285076 − *9.024334*	1106 *765*	12.22% *12.72%*	2.139238 *2.406850*
1813-Medical Helpline	1207 *852*	13.33% *12.17%*	− 35.486025 − *23.923099*	773 *309*	8.54% *5.14%*	16.285076 *9.024334*	755 *369*	8.34% *6.14%*	− 2.139238 − *2.406850*
Year									
2016	1401 *1062*	14.48% *17.66%*	− 3.6227039 − ***2.4569773***	451 *232*	4.98% *3.86%*	5.2774675 *4.9156658*	615 *369*	6.80% *6.14%*	2.1207352 *1.5869673*
2017	1605 *1208*	17.74% *20.09%*	0.5151980 *0.9909348*	401 *185*	4.43% *3.08%*	0.0250515 − *0.8053347*	627 *369*	6.93% *6.14%*	0.3147488 − *0.3226088*
2018	1767 *1343*	19.53% *22.33%*	2.9925059 ***1.3842896***	348 *171*	3.85% *2.84%*	− 5.1233044 − *3.9190487*	619 *396*	6.84% *6.59%*	− 2.3588767 − *1.2040795*
Weekday									
Week	3278 *2506*	36.22% *41.68%*	8.381580 *4.336712*	719 *384*	7.95% *6.39%*	− 3.716612 − ***1.041430***	1275 *805*	14.09% *13.39%*	3.868071 *2.999783*
Weekend	1495 *1107*	16.52% *18.41%*	− 8.381580 − *4.336712*	481 *204*	5.32% *3.39%*	3.716612 ***1.041430***	586 *329*	6.48% *5.48%*	− 3.868071 − *2.999783*
Treatment*	1152 *1091*	12.73% *18.14%*	15.023994 *9.230893*	94 *82*	1.04% *1.36%*	− 10.276163 − *6.988008*	293 *269*	3.24% *4.47%*	− 3.282012 − ***1.895487***
Thrombolysis	1041 *1016*	11.51% *16.89%*	15.887928 *10.410536*	75 *73*	0.83% *1.21%*	− 9.901901 − *6.665477*	222 *213*	2.45% *3.54%*	− 5.389991 − *4.144533*
Reperfusion	168 *146*	1.86% *2.43%*	7.3690592 *4.7924906*	8 *8*	0.09% *0.13%*	− 4.209392 − ***2.6258701***	39 *34*	0.43% *0.57%*	− 0.9567605 − *0.3453236*
Endovascular	14 *13*	0.15% *0.22%*	− 3.1609477 − *3.108757*	5 *3*	0.06% *0.05%*	− 0.5315617 − *0.343197*	25 *19*	0.28% *0.32%*	5.5483350 *5.068036*
Surgical	14 *8*	0.15% *0.13%*	− 2.6579592 − ***3.9074732***	4 *2*	0.04% *0.03%*	− 0.7672481 − *0.6252947*	19 *17*	0.21% *0.28%*	3.8412820 *5.1342675*
Thrombectomy	178 *158*	1.97% *2.63%*	6.8198305 *4.6608318*	13 *10*	0.14% *0.17%*	− 3.6134411 − ***2.4740986***	43 *37*	0.48% *0.62%*	− 0.9978678 − *0.4347634*

Characteristic	Missing criteria			Chi-Square test			Cramer’s V	Pairwise comparison	
	*N*	%	Residuals	X-Squared	*Df*	*p-*value	Estimate [Low. CI.; Upr. CI]	Adj. sig. level	Critical *z*-value
Total	1215 *678*	13.43% *11.28%*	–	–	–	–	–	–	–
Type									
Ischaemic	1129 *628*	12.48% *10.44%*	2.2363844 *2.028612*	35.762 *27.903*	3	= 8.406e−08 = *3.807e−06*	0.0629 [0.0401; 0.0819] *0.0681 [0.0397; 0.0913]*	0.00625	− 2.734269
Haemorrhagic	91 *50*	1.01% *0.83%*	− 2.2363844 − *2.028612*						
Sex									
Male	586 *333*	6.48% *5.54%*	− 3.8390984 − ***2.467792***	28.702 *15.911*	3	= 2.587e−06 = *0.01183*	0.0563 [0.0332; 0.0752] *0.0514 [0.0212; 0.0740]*	0.00625	− 2.734269
Female	629 *345*	6.95% *5.74%*	3.8390984 ***2.467792***						
EMS number									
1-1-2	81 *66*	0.90% *1.10%*	− 38.299051 − *31.542421*	2098.6 *1189.3*	3	< 2.2e−16 < *2.2e−16*	0.4816 [0.4607; 0.5020] *0.4447 [0.4191; 0.4697]*	0.00625	− 2.734269
1813-Medical Helpline	1134 *612*	12.53% *10.18%*	38.299051 *31.542421*						
Year									
2016	340 *176*	3.76% *2.93%*	− 2.4591338 − *2.7748033*	55.028 *38.962*	6	= 4.575e−10 *7.281e*−*07*	0.0551 [0.0375; 0.0674] *0.0569 [0.0344; 0.0715]*	0.0416667	− 2.86526
2017	388 *219*	4.29% *3.64%*	− 1.1524637 − *0.3789986*						
2018	487 *283*	5.38% *4.71%*	3.5109012 *3.0261982*						
Weekday									
Week	582 *347*	6.43% *5.77%*	− 13.161698 − *9.446623*	207.03 *94.963*	3	< 2.2e−16 < *2.2e*−*16*	0.1513 [0.1299; 0.1712] *0.1257 [0.0989; 0.1497]*	0.00625	− 2.734269
Weekend	633 *331*	6.99% *5.50%*	13.161698 *9.446623*						
Treatment*	123 *118*	1.36% *1.96%*	− 7.886774 − *5.385627*	260.66 *106.72*	3	< 2.23−16 < *2.2e*−*16*	0.1697 [0.1484; 0.1897] *0.1332 [0.1065; 0.1573]*	0.00625	− 2.734269
Thrombolysis	111 *110*	1.23% *1.83%*	− 7.025162 − *4.733407*	270.13 *117.16*	3	< 2.2e−16 < *2.2e*−*16*	0.1728 [0.1515; 0.1928] ***0.1396 [0.1130; 0.1638]***	0.00625	− 2.734269
Reperfusion	2 *2*	0.02% *0.03%*	− 5.4690676 − *4.5272438*	67.651 *33.67*	3	< 2.2e−16† = *1.589e*−*09†*	0.0865 [0.0644; 0.1059] *0.0748 [0.0467; 0.0982]*	0.00625	− 2.734269
Endovascular	3 *2*	0.03% *0.03%*	− 1.4200864 − *1.132402*	31.165 *25.942*	3	= 1.056e−05† = *8.998e*−*05†*	0.0587 [0.0357; 0.0777] *0.0657 [0.0370; 0.0888]*	0.00625	− 2.734269
Surgical	6 *4*	0.07% *0.07%*	0.1015225 *0.2872599*	15.579 *27.909*	3	= 0.003068† = *2.472e*−*05†*	0.0415 [0.0168; 0.0599] *0.0681 [0.0397; 0.0913]*	0.00625	− 2.734269
Thrombectomy	5 *4*	0.06% *0.07%*	− 5.2089195 − *4.3553056*	57.584 *31.176*	3	= 1.929e−12 = ***2.142e***−***08†***	0.0798 [0.0575; 0.0991] *0.0720 [0.0438; 0.0953]*	0.00625	− 2.734269

“Italic” numbers indicate the subgroup analysis of stroke patients with a time-to-call < 4.5 h. “Bold” numbers indicate the difference to the initial analysis

*A patient that received multiple treatments was considered individually for the individual treatments, but cumulated for the total number of treatments

^†^Results from the Fisher’s Exact test, due to the small number of < 5 within the group

**Fig. 2:**
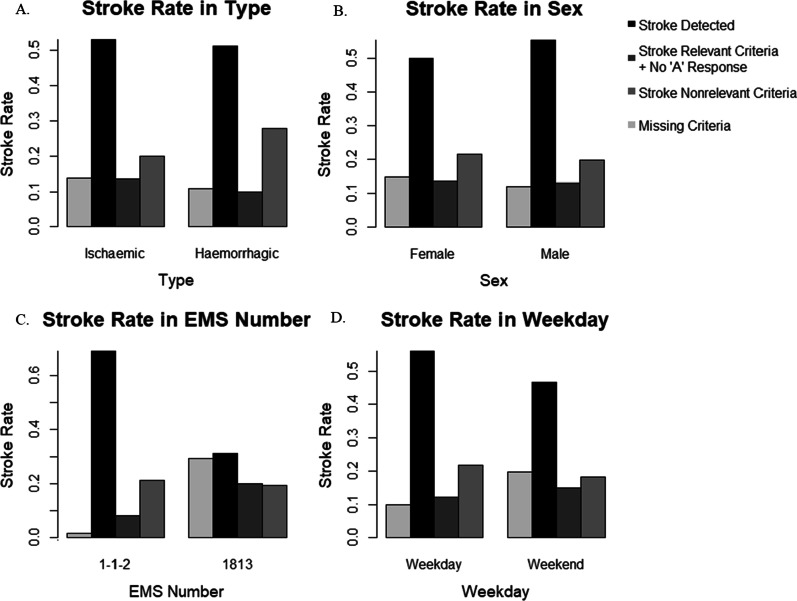
Stroke rate in type (**A**), sex (**B**), EMS number (**C**), and weekday (**D**) in EMS of the Capital Region of Denmark 2016–2018. These bar plots show the percentage of all stroke calls divided into the categories “stroke detected”, “stroke relevant criteria but no “A” response”, “stroke nonrelevant criteria”, and “missing criteria” for the characteristics type, sex, EMS number, and weekday

A positive correlation between ischaemic stroke and “stroke relevant criteria but no “A” response” and between haemorrhagic stroke and “stroke nonrelevant criteria” have been determined (Fig. 2A). Within the subgroup analysis no positive correlation between ischaemic stroke and “stroke relevant criteria but no “A” response” was observed. However, the Cramer’s V indicates, that based on the degree of freedom, the identified correlation is a weak association effect [38, 52].

Furthermore, a positive correlation exists between male and “stroke detection”. Contrarily, a negative association is recorded between female and “stroke detection” (Fig. 2B). However, based on the Cramer’s V, the strength of the correlation is very weak for the variable sex in relation to the degree of freedom [38, 52].

A change in correlation throughout the years 2016–2018 was reported. While the “stroke detection” in 2016 has a negative association, 2018 shows a positive association. Conversely, 2016 indicates a positive correlation to “stroke relevant criteria but no “A” response”, while a negative correlation is identified in 2018, The correlations reported for the category “stroke detection” could however not be determined within the subgroup analysis. The association reported is considered weak, based on the results of the Cramer’s V and under consideration of the respected degree of freedom [38, 52].

Furthermore, the weekdays (Monday–Friday) are in positive correlation with “stroke detection” and “stroke nonrelevant criteria”, while the weekend (Saturday–Sunday) is in positive correlation with “stroke relevant criteria but no “A” response” and “missing criteria” (Fig. 2D). Conversely, in the subgroup analysis no correlation between “stroke relevant criteria but no “A” response” and weekday or weekend could be detected. The reported association is weak based on the degree of freedom [38, 52]. On the weekend 53.11% of all EMS stroke contacts were through 1813, compared to 37.1% within the week.

For the overall treatment, the analysis identified a positive association with regards to “stroke detection”. Additionally, for thrombolysis, a positive correlation with “stroke detection” was determined (Fig. 4A). The Cramer’s V indicates that, based on the degree of freedom, the strength of the identified correlation is weak for the overall treatment and moderate for thrombolysis [38, 52]. Within the subgroup analysis similar results could be observed, however for thrombolysis only a weak strength of association was identified.

Considering the time-to-call, 75.7% of all “stroke detected” calls were within 4.5 h after stroke onset. Comparably within the category “stroke nonrelevant criteria” 60.93%, “stroke relevant criteria but no “A” response” 49% and “missing criteria” 55.8% of the calls were within 4.5 h after stroke onset (Table 3).

**Table 3:** Time-to-call in EMS of the Capital Region of Denmark 2016–2018

Category	Time-to-call		
	Within 4.5 h (%)	Within 24 h (%)	Mean (Min.; Q1; Median; Q3; Max.)
Stroke detected	75.70	94.93	5.65 (0.00045; 0.312; 1.233; 4.296; 165.861)
Stroke relevant criteria + no “A” response	49.00	80.42	16.22 (0.0053; 1.019; 4.721; 18.914; 167.646)
Stroke non relevant criteria	60.93	85.06	14.07 (0.0016; 0.665; 2.451; 11.289; 167.377)
Missing criteria	55.80	83.62	14.16 (0.011; 0.995; 3.391; 12.514; 167.456)

Time of onset is based on the patients recall of symptom onset and thus might be no exact time of onset

The Kruskal–Wallis test indicates a statistically significant difference in stroke detection with regards to age (Table 4). While the Wilcoxon–Mann–Whitney post-hoc test with a Bonferroni-Holm correction additionally determines that a statistically significant difference in age between “stroke detected” and “stroke relevant criteria but no “A” response” as well as “missing criteria” and between “stroke relevant criteria but no “A” response” and “stroke nonrelevant criteria” as well as “missing criteria” exists. When considering the mean age of “missing criteria” (72.53), “stroke detection” (71.4), “stroke nonrelevant criteria” (71.08), and “stroke relevant criteria but no “A” response” (69.87), the latter group is statistically significantly younger than the three previously named groups. Additionally, the stroke patients with “missing criteria” are statistically significantly older than the stroke patients detected by the EMD. Comparably, within the subgroup analysis only a statistically significant difference in age was determined between “missing criteria” and “stroke relevant criteria but no “A” response” as well as “stroke nonrelevant criteria” (Table 4). However, the mean age decreases in following direction “missing criteria” (71.9), “stroke detection” (71.06), “stroke non relevant criteria” (69.96), and “stroke relevant criteria but no “A” response” (69.94).

**Table 4:** Outcome variance-analysis

Characteristics	Stroke detected		Stroke not detected				Missing criteria	
	Stroke relevant criteria + “A” response		Stroke relevant criteria + no “A” response		Stroke nonrelevant criteria			
	*N*	Mean [Min.|Q1|Median|Q3|Max.]	*N*	Mean [Min.|Q1|Median|Q3|Max.]	*N*	Mean [Min.|Q1|Median|Q3|Max.]	*N*	Mean [Min.|Q1|Median|Q3|Max.]
Age	4773 *3613*	71.4 [18|63|73|81|104] *71.06 [19|62|72|81|104]*	1200 *588*	69.87 [21|61|71|80|101] *69.94 [21|61|71|80|101]*	1816 *1134*	71.08 [21|63|73|82|101] *69.96 [21|60|72|81|101]*	1215 *678*	72.53 [19|63|74|83|102] *71.90 [19|62|73|83|102]*
Time of day	4773 *3613*	12.82 [0|9|12|17|23] *12.93* *[0|9|13|17|23]*	1200 *588*	13.55 [0|10|14|17|23] *13.36* *[0|10|13|17|23]*	1816 *1134*	12.97 [0|9|13|17|23] *12.60* *[0|9|12|16|23]*	1215 *678*	13.83 [0|10|15|18|23] *13.56* *[0|10|15|18|23]*

Characteristics	Kruskal–Wallis test			Wilcoxon–Mann–Whitney (+ Bonferroni–Holm Correction)			
	Kruskal–Wallis-*X*^*2*^	*Df*	*p*-value				
Age	29.226 *13.944*	3	2.00e−06 *0.002983*		Stroke Detected	Stroke relevant criteria + no “A” response	Stroke non-relevant criteria
				Stroke relevant criteria + no “A” response	0.0017 ***0.1368***	–	–
				Stroke non-relevant criteria	1.000 *0.8197*	0.0055 *1.0000*	–
				Missing criteria	0.0068 ***0.1551***	8.7e−07 *0.0052*	0.0543 ***0.0273***
Time of day	63.455 *24.8*	3	1.073e−13 *1.7e−05*		Stroke Detected	Stroke relevant criteria + no “A” response	Stroke non-relevant criteria
				Stroke relevant criteria + no “A” response	2.8e−05 ***0.39660***	–	–
				Stroke non-relevant criteria	0.934 *0.68081*	0.019 *0.04429*	–
				Missing criteria	7.1e−12 *0.00037*	0.055 *0.62118*	3.5e−07 *2.5e−05*

“Italic” numbers indicate the subgroup analysis of stroke patients with a time-to-call < 4.5 h. “Bold” numbers indicate the difference to the initial analysis

**Fig. 3:**
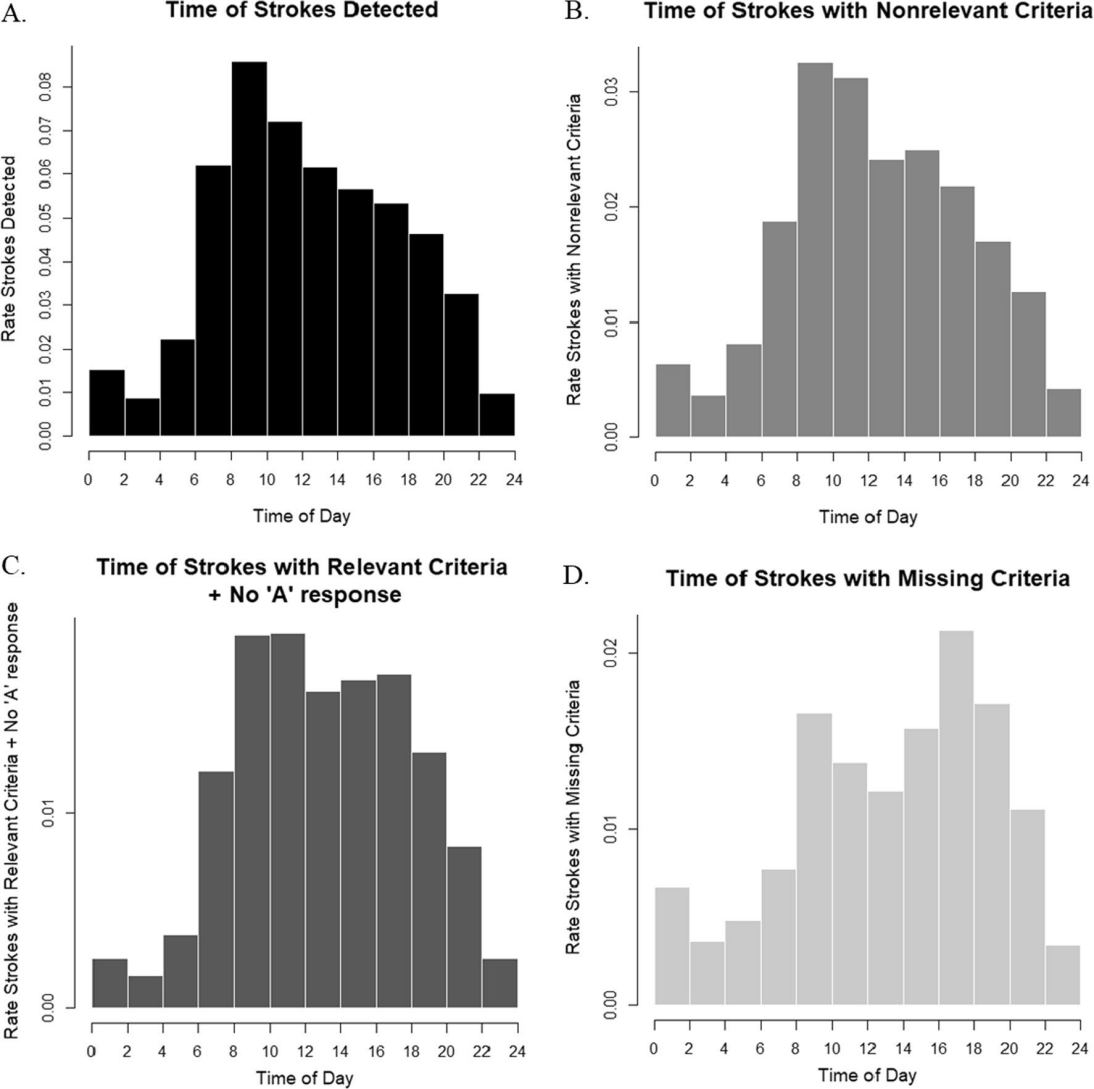
Histogram—time of strokes within the categories “Strokes Detected” (**A**), “Strokes with Nonrelevant Criteria” (**B**), “Strokes with Relevant Criteria but no “A” Response” (**C**), and “Strokes with Missing Criteria” (**D**) in EMS of the Capital Region of Denmark 2016–2018. These histograms show the rate of stroke calls throughout the day for all four categories based on all stroke calls

For the time of day, a statistically significant difference in stroke detection was identified (Table 4). A statistically significant difference in the time of the call between “stroke relevant criteria but no “A” response” and “stroke detection” as well as “stroke nonrelevant criteria” has been determined through the Wilcoxon–Mann–Whitney post-hoc test with a Bonferroni-Holm correction. Additionally, a statistically significant difference in time of the EMS call between “missing criteria”, “stroke detected”, and “stroke nonrelevant criteria” has been detected. Comparably, no statistically significant difference in time of day between “stroke detection” and “stroke relevant criteria but no “A” response” could be seen in the subgroup analysis (Table 4). It can be observed that the groups “stroke detected” and “stroke nonrelevant criteria” have their peak before 10 a.m. and then steadily decrease (Fig. 3A + B). Comparatively, the group “stroke nonrelevant criteria, but no “A” response” decreases only slightly after 12 p.m. but stays on a relatively high level until 6 p.m. after which the number of strokes with “stroke relevant criteria but no “A” response” decrease (Fig. 3C). When considering the histogram of “time of strokes with missing criteria”, two peaks can be observed, one in the morning and one in the afternoon (Fig. 3D).

**Fig. 4:**
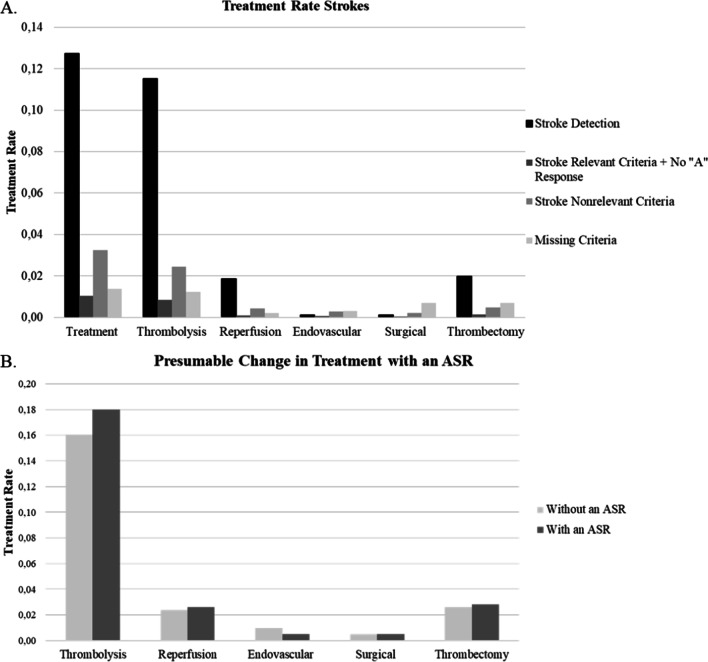
Treatment Rate of Strokes (**A**) and Presumable Change in Treatment Rate with an ASR (**B**). **A** Proportion of stroke patients treated with the considered treatment options divided in the four categories. **B** Presumable change in the proportion of the different treatment options through an ASR

Based on the results of the statistical analyses, calculations on how an ASR in the EMS could have potentially impacted the stroke detection in the years 2016–2018 have been performed. This is under the condition that an ASR would improve stroke detection similarly as has been shown for the detection of OHCA by the research of Blomberg et al. For this calculation the strokes with “missing criteria” will be treated as if they are not influenced by the ASR. Presumably, the stroke detection rate in the EMS Copenhagen could rise to 61.19% [24]. Therefore, a supporting ASR tool could assumingly have increased the amount of strokes detected by 764 (16%) from 4773 to 5537 (*n* = 9049) in the years 2016–2018. Additionally, assuming that the EMS contact was within the appropriate time-to-treatment, the thrombolysis rate among stroke patients could increase from 16 to 18%. Comparatively, the reperfusion rate could increase from 2.4 to 2.6%, the thrombectomy rate from 2.6 to 2.8%, and the surgical treatment rate from 0.48 to 0.49%. However, based on the data analysis and under the named conditions, the endovascular treatment rate would decrease from 0.52 to 0.49% (Fig. 4B). Under consideration of the time-to-call (4.5 h), the subgroup analysis indicated that the stroke detection rate within this subpopulation the stroke detection rate with an ASR could increase to 69.7% and thus increase the thrombolysis rate within the stroke patients calling within time-to-treatment of thrombolysis by 5%. Contrarily, the amount of endovascular treatment would have presumably decreased by 14%, while surgical treatment would have decreased by 16% if an ASR would have been used for stroke recognition within the years 2016–2018.

## Discussion

The analysis suggests that a significant number of stroke calls are not detected as strokes (33.83%) within the 1-1-2 and 1813 emergency medical contact points. Considering the positive effects stroke recognition at the EMS takes on the stroke related outcome, the improvement of stroke detection at the EMS is crucial [14–16, 18, 19]. This research suggests the usage of an ASR, based on the model of CORTI AI for OHCA, to increase stroke recognition at the EMS from 52.75 to 61.19%. This increased detection rate through an ASR might decrease the number of multiple EMS calls for stroke patients, due to an earlier detection of the stroke and an accurate response within the first call. However, further research to determine the reason for multiple EMS calls would be necessary. Based on the condition that the stroke detection rate would increase by the same amount as the OHCA detection rate increased through CORTI AI, the rate of stroke patients treated with thrombolysis will rise by 5% within the group of stroke patients calling within time-to-treatment for thrombolysis [24, 54]. Additionally, the ASR might lead to an increase in thrombectomy of 8%, reperfusion of 8%, and surgical treatment of 2%. However, these increasing rates for thrombectomy, reperfusion and surgical treatment are to be viewed with caution. While the strength of the identified correlation between stroke recognition and treatment is moderate for thrombolysis, it is weak or very weak for the other treatment options. Additionally, the calculations have been made based on theoretical background and under the condition that the patients call the EMS within the treatment specific time-to-treatment. While 66.45% of all EMS contacts are within time-to-treatment of thrombolysis (4.5 h), 89.46% are within 24 h after stroke onset. Mosley et al. [55] confirm these findings by reporting that less than 50% of the stroke related calls were within 60 min after stroke onset [55]. In the future, a prospective study on the change in treatment through an increase in stroke detection would be interesting.

The described results suggest, that with an increased stroke detection at the EMS, the rate of stroke patients receiving endovascular treatment might decrease. The subgroup analysis of stroke patients with a time-to-call < 4.5 h showed a decrease in endovascular and surgical treatment. Nonetheless, this must be considered with caution, since endovascular treatment is regarded as an alternative for unsuccessful thrombolysis or patients not eligible for thrombolysis and surgical treatment is only carried out occasionally and under selected circumstances [56, 57]. While the time-to-treatment for thrombolysis is 4.5 h, endovascular treatment can be received within six to eight hours after stroke onset [56, 58]. Thus, patients who are not eligible for thrombolysis due to the closure of the window of time-to-treatment might receive endovascular treatment. In contrast, other reasons influence the choice of endovascular treatment [56]. Additionally, due to the low number of endovascular (*n* = 47) and surgical (*n* = 43) treatment, the results of these categories cannot be emphasized, but further research with a larger number of stroke patients treated with endovascular and surgical treatment would be necessary to draw conclusions [59].

Moreover, several additional factors, like stroke detection by the caller, recognition by the paramedic on scene, pre-conditions, and personal characteristics impact the stroke patients eligibility for treatment [14, 60]. Jones et al. [61] determined that symptoms like speech problems as well as posterior circulation symptoms were least likely to be recognised as stroke related. Further research on the beforementioned connections as well as on mortality and on the score of the modified ranking scale, which defines a patients clinically discrete disability caused by a stroke on a scale of seven levels, would be helpful in order to draw precise and grounded conclusions on the effect of EMS stroke detection on patient outcome [62, 63]. Nonetheless, stroke detection by the EMS might impact the treatment, specifically thrombolysis. The relevance of an ASR for stroke detection at the EMS is underlined by the substantial amount (49% of “stroke relevant criteria but no “A” response” and 60.93% of “stroke nonrelevant criteria”) of calls within time-to-treatment for thrombolysis in the categories “strokes not detected”.

Based on the results of the analysis, it can be argued that the ASR could specifically impact the detection of those characteristics with a negative correlation to “stroke detected” or a positive correlation to one of the categories within “strokes not detected”.

The analysis indicates that an improvement of stroke detection is particularly important for calls to the 1813 Medical Helpline, due to the observed negative correlation of stroke detection within 1813-calls. Thus, the ASR should be used for both access numbers 1813 and 1-1-2. The negative correlation may be influenced by non-recognition of atypical stroke symptoms by the caller, thus the 1813 instead of the 1-1-2 is called [64]. However, for validation further research is needed.

When training the ASR specific attention should be placed on haemorrhagic strokes, due to the positive correlation between haemorrhagic strokes and “stroke nonrelevant criteria” and the small representation of haemorrhagic strokes (9.22% of all strokes). Several authors argue, that it is particularly important to take into account underrepresented groups, e.g. haemorrhagic stroke patients (*n* = 834) and patients with “stroke nonrelevant criteria” (*n* = 1215), when training an ASR, in order to avoid a bias, that could possibly cause an erroneous stroke detection algorithm [65, 66]. An ASR could also positively influence the stroke detection rate of females, due to the negative correlation to stroke detection. Lisabeth et al. [67] and Rathore et al. [68] support this finding by describing, that women reported a larger amount of non-traditional stroke symptoms.

In the data analysis a negative correlation between stroke detection and weekends was determined, hence the ASR for stroke detection could particularly improve the stroke detection on weekends. A possible explanation could be, that on weekends 53.11% of all stroke related EMS calls are to the 1813, while within the week only 37.1% are to the 1813. As argued before, 1813-calls might entail more atypical stroke symptoms not detected compared to the 1-1-2, resulting in a decline of the detection rate on the weekends [64].

Interestingly, stroke patients within the group “stroke relevant criteria but no “A” response” are significantly younger than the patients within the other groups. Considering the research by Singhal et al. [69], detection of stroke among younger patients, is challenging due to infrequency in comparison to stroke mimics and missing awareness among the general population as well as the EMDs. This might result in an EMS contact outside of the window of time-to-treatment or missing recognition of severity and thus in no “A” response. This is strengthened by “stroke relevant criteria but no “A” response” having the lowest proportion of calls (49%) within the time-to-treatment for thrombolysis. This reasoning is also supported by the subgroup analysis showing no statistically significant difference in age between the categories “stroke detection” and “strokes not detected”.

The category “stroke relevant criteria but no “A” response”, has a different distribution throughout the time of day, compared to strokes detected and strokes with non-relevant criteria. While the latter have a peak between 8 a.m. and 10 a.m. and thereafter steadily decrease, the beforenamed category is comparatively steady between 8 a.m. and 6 p.m. The peak of stroke relevant criteria in the morning might be due to the so called “wake-up stroke”, for which EMDs have a high awareness, since one out of five strokes is a “wake-up stroke” [70]. Comparably, in the afternoon a greater diversity among emergency calls occurs, which might result in a higher difficulty to detect strokes [71]. For these calls an ASR supporting the EMD in the stroke detection would be useful to detect and send the correct response. Due to the subgroup analysis identifying no statistically significant difference in time of day between “stroke detection” and “strokes not detected” an ASR would be relevant for increasing stroke detection throughout the whole day. The steady number of calls with “stroke relevant criteria but no “A” response” might be caused by a delayed emergency call and despite the stroke detection by the dispatcher, but due to the closure of the window of time-to-treatment, no “A” response. This argument is supported by the outcome of the subgroup analysis, showing no statistically significant difference in time of day between “stroke detection” and “stroke relevant criteria but no “A” response”. Further research to conduct the reason for stroke relevant criteria but no “A” response is necessary. The “missing criteria” show two peaks throughout the day, between 8 a.m. and 10 a.m. as well as between 4 p.m. and 6 p.m. These peaks can be explained by the majority of “missing criteria” within 1813 calls, and the increased amount of 1813 calls between 8 a.m. and 10 a.m. on the weekends and between 4 p.m. and 6 p.m. during the week, due to its mission as out-of-hours general practitioner [13]. However, additional factors that might be influenced by an ASR, were not considered in this study.

Due to a significant increase of stroke detection throughout the years 2016–2018, as shown in our analysis, it might be argued that no further technical support might be necessary to improve stroke detection. However, since a significant decrease has only been seen within the group “stroke relevant criteria but no “A” response”, but not within the group “stroke nonrelevant criteria”, this argument can be discarded due to the ASR presumably impacting the recognition of strokes with currently “stroke nonrelevant criteria”, by increasing the detection of stroke symptoms and thus indicating “stroke relevant criteria”. Additionally, the subgroup analysis indicating no statistically significant increase in stroke detection throughout the years 2016–2018 supports the need of an ASR for improving stroke recognition by the EMDs. The increase in stroke detection seen for 2016–2018 might be influenced by the publication by Viereck et al. [15] in 2016 on the recognition of strokes through EMDs, after which small changes have been made in the algorithm of the 1-1-2. Another reason for the change in stroke detection throughout the years 2016–2018, could be the results of a research conducted at the University of Kentucky Stroke Center impacting the stroke recognition campaign, “FAST” (Face, Arm, Speech, Time) to “BE-FAST” (Balance, Eyes, Face, Arm, Speech, Time) in 2017, through including visual symptoms on stroke [72]. This revision might have led to an increasing sensibility for strokes within the population possibly resulting in a clearer expression of the symptoms to the EMS and an increasing sensibility of EMDs for stroke related symptoms [72].

The question arises, whether other options could increase stroke detection by EMS call-takers. Past research analysed the influence of educational training modules as well as stroke recognition scales and protocols, such as the “FAST”-Tool [17, 73–75]. However, Oostema et al. [73] reported, that the increase in stroke recognition after an educational intervention was limited to three months and might increase the rate of false positive stroke detection due to a higher sensibility to symptoms related to stroke [73, 76]. Additionally, the systematic review by Oostema et al. [17] discovered, that the correct usage of the scales and protocols has not been analysed in the included studies, resulting in lacking security of the right usage. It is to be mentioned, that educational programmes for EMDs might increase the rate of false positive stroke detection due to a higher sensibility to symptoms related to stroke [76].

Like the correct use and acceptance of scales and protocols, the acceptance and adoption of the ASR into the EMS call by the EMD, is relevant for its effect on stroke detection. Blomberg et al. [77] reported a lack of compliance with the suggestions of CORTI AI by the EMDs, which resulted in no increase of OHCA detection within the EMS Copenhagen. Considering the results of educational interventions, the introduction of an ASR for strokes at the EMS could be accompanied by, for example educational interventions addressing challenges in the uptake of the ASR, in order to ensure the effect of the ASR [73–75, 77]. The European Institute of Innovation and Technology (EIT) Health states that to improve the uptake and effect of AI in healthcare, investments in the education of healthcare workers to ensure digital literacy, the exchange of best practice in the field of AI in healthcare throughout the EU and improvement of collaboration is essential [78].

Despite the lack in compliance with the ASR and thus the limitation of the effect, no sole usage of an ASR should be aimed for, due to possible input and algorithm bias as well as the missing consideration of the emotional component [79, 80]. In summary, the combination of an ASR with a well-trained human professional can substantially increase the number of correctly detected strokes [24, 25].

### Limitations

The definition of stroke detection as “stroke relevant criteria” and an “A” response, might not represent all the strokes detected within the EMS. Possibly, strokes were detected within the category “stroke relevant criteria but no “A” response”, and still, due to the closure of the window of time-to-treatment no “A” response was sent. For those cases obviously, an ASR would not impact the stroke detection. Contrarily, strokes might have been detected within the category “missing criteria”, but no criteria were indicated within the system, yet an “A” response had been sent as the correct stroke response. Likewise, possibly “stroke nonrelevant” criteria were indicated within the system, but the EMD recognised the stroke and sent an “A” response. Due to the definition of stroke response within the EMS Copenhagen, the proxy of “stroke relevant criteria” and “A” response was considered the most accurate to define stroke detection for this research.

Another limitation of the stroke related emergency calls is, that all EMS calls seven days prior and post stroke were included within this study, even if the emergency call was not related to the stroke of the patient but was due to another medical issue. However, research has shown that strokes typically impact the health of the patient significantly, through post-stroke and pre-stroke symptoms, thus the number EMS contacts of stroke patients not related to the stroke might be comparably small [36, 67]. The choice to include stroke calls seven days prior and post stroke could be affecting the response made by the medical dispatcher, depending on the time of symptom onset named within the call and thereby diminish the effect of the outcome. Unfortunately, the data on time of symptom onset is not documented and thus not available and must therefore be considered a blind spot within this research. Additionally, the subgroup and time-to-call analysis is limited due to the determination of stroke onset within the patient-doctor consultation based on the patients recall of time of symptom onset. Thus, the possibility of recall bias needs to be considered in the interpretation of the results [81, 82].

The internal validity, which is described as to which extent the study accurately measures the concept, might be also limited due to the assumption, that an ASR for stroke has the same effect on the increase of detection at the EMS, as CORTI AI on OHCA, since OHCA symptoms are more specific compared to stroke symptoms [33, 83–86]. Thus, the possibility of stroke mimics, which are defined as disorders showing stroke symptoms, such as for example brain tumours, metabolic disorders, or migraines, and are diagnosed as strokes are likelier than false positive OHCA [85]. This is supported by the research by Watkins et al. [84] detecting a specificity of 99.4% within OHCA, while according to Hatzitolios et al. [85] 5% and to Hosseininezhad and Sohrabnejad [86] 14.9% of all stroke-like symptoms are stroke mimics. However, since CORTI AI for OHCA is, to the researcher’s knowledge, the only ASR within an EMS context, the presumable increase of 16% based on CORTI AI was chosen. Hence, this limitation must be considered when referring to the presumable increase in stroke detection, especially since Blomberg et al. [24] reported a decrease in specificity for OHCA detection with the ASR from 98.8 to 97.3% (*p* < 0.001). Under consideration of the beforenamed rate of stroke mimics, the decrease in specificity of stroke detection might be higher with an ASR compared to the decrease in specificity of OHCA. Further research on the topic of specificity in stroke detection through ASR should be performed to elaborately address this point and to discuss possible mitigation strategies.

The transferability to the population of Denmark would need further research, since the results conducted for the Capital Region of Denmark, with the specialty of the 1813, might not be transferable to the entirety of Denmark [87]. Additionally, the transferability to other countries might be limited, due to country specific EMS and population characteristics. Thus, the assessment of transferability on superordinate level using for example the PIET-T Model might be helpful [88]. Because of the mentioned limiting factors, the results of this study should be interpreted with caution and considered as directing and indicating further research fields.

## Conclusion

An ASR can presumably improve the recognition of stroke. Based on the results of this research, an intervention to increase stroke recognition is important for the EMS Copenhagen, specifically among females, younger stroke patients, within the 1813-Medical Helpline, and on weekends. Under consideration of the beforenamed conditions and limitations, an ASR could have a positive effect on stroke detection, and thereafter on stroke treatment, specifically on thrombolysis.

## Data Availability

The data that support the findings of this study are available from Emergency Medical Services, Capital Region of Denmark, Denmark but restrictions apply to the availability of these data, which were used under license for the current study, and so are not publicly available. Data are however available from the authors upon reasonable request and with permission of Emergency Medical Services, Capital Region of Denmark, Denmark.

## Abbreviations

1813: 1813-Medical Helpline
AI: Artificial intelligence
ASR: Automatic speech recognition software
CI: Confidence interval
DALY: Disability-adjusted life year
EMD: Emergency Medical Dispatcher
EMS: Emergency Medical Service
OHCA: Out-of-Hospital cardiac arrest
